# Prevalence of lipase producer *Aspergillusniger* in nuts and anti-biofilm efficacy of its crude lipase against some human pathogenic bacteria

**DOI:** 10.1038/s41598-021-87079-0

**Published:** 2021-04-12

**Authors:** Asmaa S. Yassein, Mohamed M. Hassan, Rokaia B. Elamary

**Affiliations:** 1 Faculty of Science, Botany and Microbiology Department, South Valley University, Qena, 83523 Egypt; 2 Faculty of Science, Department of Biology, Taif University, P.O.Box 11099, Taif, 21944 Kingdom of Saudi Arabia

**Keywords:** Biological techniques, Biotechnology, Microbiology, Molecular biology

## Abstract

Nuts are the natural source of healthy lipids, proteins, and omega-3. They are susceptible to fungal and mycotoxins contamination because of their high nutritional value. Twenty-five species comprising 12 genera were isolated from 80 samples of dried fruits and nuts using the dilution plate method. Peanut recorded the highest level of contamination followed by coconut; almond and raisin were the lowest. *Aspergillus* was the most prevalent genus and *A.* *niger*, was the most dominant species. The morphological identification of the selected *A.* *niger* isolates as they were detected in high frequency of occurrence was confirmed by using 18SrRNA sequence. Ochratoxin biosynthesis gene *Aopks* was detected in the tested isolates. Lipase production by the selected *A.* *niger* isolates was determined with enzyme activity index (EAI) ranging from 2.02 to 3.28. *A.* *niger*-26 was the highest lipase producer with enzyme activity of 0.6 ± 0.1 U/ml by the trimetric method. *Lip2* gene was also detected in the tested isolates. Finally, the antibacterial and antibiofilm efficiency of crude lipase against some human pathogens was monitored. Results exhibited great antibacterial efficacy with minimum bactericidal concentration (MBC) of 20 to 40 µl/100 µl against *Escherichia* *coli*, *Pseudomonas* *aeruginosa*, *Proteus* *mirabilis*, and Methicillin-resistant *Staphylococcus* *aureus* (MRSA). Interestingly, significant anti-biofilm efficacy with inhibition percentages of 95.3, 74.9, 77.1 and 93.6% was observed against the tested pathogens, respectively.

## Introduction

Dried fruits and nuts are enriched source of healthy fatty acids, protein, potassium, dietary fibers and bioactive compounds^1^. They protect the mankind from the risks of obesity, cardiovascular illnesses, type 2 diabete and hypertension^2, 3^. *Alternaria*, *Aspergillus,* *Candida,* *Fusarium,* *Mucor*, *Rhizopus,* *Penicillium*, *Trichoderma*, and *Cladosporium* are the most common genera causing nuts spoilage, and their ingestion may cause mycoses especially in immunocompromised patients^4,5^.

Many species of fungi produce mycotoxins, secondary metabolites of small molecular sizes (MW < 300) that are toxic to humans and animals cause mycotoxicoses when ingested, leading to cancer and liver diseases^6, 7^. A total of 145 secondary metabolites were discovered in *Aspergillus* section Nigri; among them are ochratoxin A (OTA), which are the most toxic to humans and animals^5^. OTA is the causative agent of Balkan endemic nephropathy, urothelial tumors, and testicular cancer in humans^8–10^.

Lipase, an enzyme belonging to the serine hydrolase class, catalyzes the hydrolysis of fats and oils to glycerol and fatty acids without requiring cofactors^11^. Fungi are considered the best producers of lipase among all microorganisms, especially, *Aspergillus* *niger* which was generally recognized as safe (GRAS) by Food and Drug Administration (FDA) in the United States^12^. Lipase has a wide range of industrial applications, such as in the food industry, as detergent additives, pharmaceutical industry, and biofuel production; therefore, the universal demand for the lipase enzyme is increasing^13^.

Enzymes including isomerases, lyases, oxidoreductases, transferases, esterases and hydrolases have been reported to induce antibacterial efficacy^14^. Lipase is a hydrolytic enzyme, has antimicrobial and antifouling properties^15^. However, its mode of action and its effects in most of the cases have not been clarified fully^14^.

Biofilm is a complex medium involving live and dead bacterial cells, exopolysaccharides, proteins and carbohydrates on a material surface with a serious problem in biomedical applications^16^. Several steps were involved in biofilms development beginning with surface adherence, microcolony formation, maturation and finally detachment stages^17^. Biofilms protect pathogenic bacteria from human immune system, antibiotics and severe environmental conditions^18^. Several bio-active and chemically synthesized compounds have been performed to suppress biofilm formation by pathogenic bacteria^19–22^. Revitalize aminoglycosides also have been used to inhibit biofilm and pathogenic bacterial infections^23^. Attenuating motility properties can be considered as highly potential for controlling biofilm formation since attachment was one of the main steps in biofilm formation^24,25^. Using enzymes is also a good policy for biofilm elimination because enzymes are rabidly eco-friendly and degradable^26,27^.

This study was established for the isolation and identification of mycobiota associated with four kinds of nuts, determination the ochratoxigenic potential of some *A.* *niger* isolates, their lipolytic activity, and finally studying the ability of crude lipase from *A.* *niger* to inhibit the growth and biofilm formation of some human pathogens.

## Results

### Mycobiota contaminating nuts

Twenty-five fungal species comprising 12 genera were obtained from the 80 tested samples of nuts by using dilution plate method. *Aspergillus* was the most prevalent genus as it was isolated from 100% of the samples. *Penicillium* was the second genus in frequency as it was isolated from 62.5% of total samples. From the above genera *A.* *niger*, *A.* *flavus*, *P.* *chrysogenum* and *P.* *oxalicum* were the most frequent species (Table 1). *Rhizopus* *stolonifer* was isolated from 60% of peanut, 45% of almond and 35% of raisin but not detected in coconut. The remaining genera and species were isolated in rare frequency accounting collectively 1.59 × 10^3^ CFU/g as illustrated in Table 1.

**Table 1:** Colony forming units (CFU/g), percentage (%C), frequency (F%) and number of cases of isolation (NCI) of mycobiota contaminating nuts.

Fungal genera and species	Peanut				Almond				Coconut				Raisin			
	Fungal count (CFU/g × 10^3^)	%C	F%	NCI	Fungal count CFU/g × 10^3^	%C	F%	NCI	Fungal count (CFU/g × 10^3^)	%C	% F	NCI	Fungal count (CFU/g × 10^3^)	%C	F%	NCI
*Acremonium* *hyalinulum*	0.06	0.3%	5	1	–	–	–	–	**-**	**-**	**-**	**-**	**-**	**-**	**-**	**-**
*Alternaria* *alternata*	0.18	0.9%	5	1	0.03	0.18%	5	1	**-**	**-**	**-**	**-**	**-**	**-**	**-**	**-**
*Aspergillus*	15.68	78.75%	100	20	15.12	89.68%	100	20	11.67	77.18%	100	20	6.39	68.49%	100	20
*A.* *fumigatus*	0.65	3.26%	15	3	0.06	0.36%	10	2	0.42	2.78%	30	6	0.51	5.46%	45	9
*A.flavus*	1.53	7.68%	55	11	4.2	24.91%	65	13	5.85	38.69%	100	20	3.81	40.84%	95	19
*A.niger*	12.99	65.2%	100	20	10.83	64.23%	100	20	4.92	32.54%	75	15	2.07	22.19%	70	14
*A.sydowii*	–	–	–	–	0.03	0.18%	–	–	0.12	0.79%	10	2	–	–	–	–
*A.terreus*	0.51	2.56%	10	2	–	–	–	–	0.12	0.79%	10	2	–	–	–	–
*A.ustus*	–	–	–	–	–	–	–	–	0.24	1.58%	5	1	–	–	–	–
*Emericella* *nidulans*	0.24	1.2%	5	1	–	–	–	–	0.03	0.21%	5	1	0.12	1.29%	5	1
*Eurotium* *amstelodami*	–	–	–	–	0.03	0.18%	5	1	–	–	–	–	–	–	–	–
*E.* *chevalieri*	0.12	0.6%	5	1	–	–	–	–	–	–	–	–	–	–	–	–
*Fusarium* *dimerum*	0.12	0.6%	5	1	–	–	–	–	0.09	0.6%	10	2	–	–	–	–
*F.* *solani*	0.12	0.6%	10	2	–	–	–	–	–	–	–	–	–	–	–	–
*Mucor* *circinelloides*	–	–	–	–	–	–	–	–	–	–	–	–	0.24	2.57%	15	3
*Paecilomyces* *variotii*	0.12	0.6%	5	1	–	–	–	–	–	–	–	–	–	–	–	–
*Penicillium*	1.5	7.53	55	11	0.57	3.38%	40	8	2.01	13.29%	80	16	2.04	21.86%	75	15
*P.* *aurantiogriseum*	0.06	0.3%	5	1	–	–	–	–	–	–	–	–	–		–	–
*P.chrysogenum*	0.69	3.47%	25	5	0.36	2.14%	35	7	0.57	3.77%	30	6	0.54	5.79%	35	7
*P.duclauxii*	0.12	0.6%	5	1	–	–	–	–	–	–	–	–	–		–	–
*P.funiculosum*	0.06	0.3%	5	1	–	–	–	–	–	–	–	–	0.06	0.64%	5	1
*P.* *oxalicum*	0.39	1.96%	20	4	0.21	1.25%	20	4	1.44	9.52%	75	15	1.44	15.43%	70	14
*P.purpurogenum*	0.12	0.6%	5	1	–	–	–	–	–	–	–	–	–		–	–
*P.variabile*	0.06	0.3%	5	1	–	–	–	–	–	–	–	–	–		–	–
*Rhizopus* *stolonifer*	1.62	8.14%	60	12	1.11	6.58%	45	9	–	–	–	–	0.54	5.79%	35	7
*Scyatlidium* *lignicola*	0.06	0.3%	5	1	–	–	–	–	1.32	8.73%	75	15	–	–	–	–
*Stachybotrys* *atra*	0.06	0.3%	5	1	–	–	–	–	–	–	–	–	–	–	–	–
Sterile mycelia	0.03	0.15%	5	1	–	–	–	–	–	–	–	–	–	–	–	–
Total	19.91	100%			16.86	100%			15.12	100%			9.33	100%		
Number of genera = 12	11				5				5				5			
Number of species = 25	21				9				11				9			

CFU/g: Colony forming unit per gm of 80 samples of peanut, almond, coconut and raisin (20 of each) on rose Bengal chloramphenicol agar medium (RBCA). %C: Percentage of each isolate to the total isolates for each type of nuts. F%: Frequency of each isolate. NCI: number of cases of isolation of each isolate out of 20 sample of peanut, almond, coconut and raisin.

### Multiple alignment of different *A*.* niger* isolates

**Figure 1:**
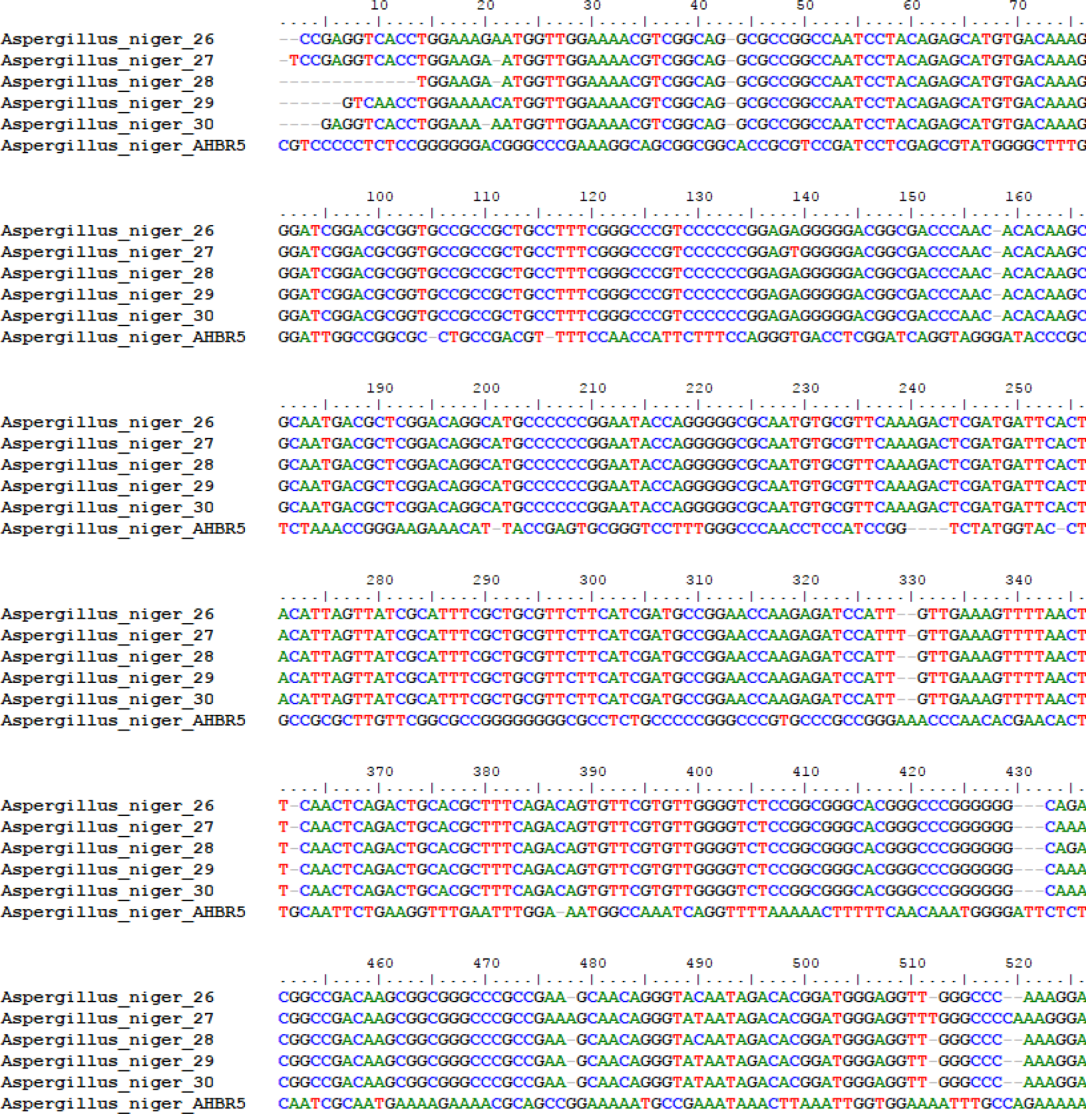
Comparison alignment of nucleotide sequences of ITS region in five *A.* *niger* isolates with different relation nucleotide sequences of some strains at NCBI database using BioEdit program.

**Figure 2:**
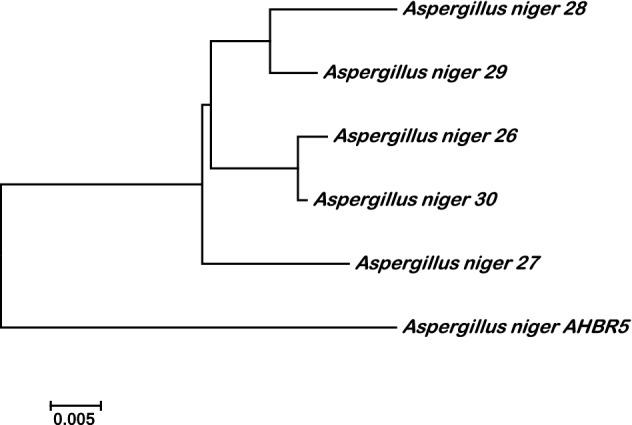
Phylogenetic tree and relationship among five strains of *A.* *niger* compared with some *A.* *niger* strains at NCBI.

The 5.8S gene in rDNA sequences were subjected to multiple alignments using the BioEdit program (http://www.mbio.ncsu.edu/BioEdit/page2.html). Among the five isolates of *A.* *niger*, 5.8S gene nucleotide sequences showed 98% similarity all strains. When the sequences were aligned with the database sequences, they showed 95% similarity with *A.* *niger* strain AHBR5, except the *A.* *niger*-27 similarity sequence, which shared 98% similarity with *A.* *niger* strain AHBR5 (Fig. 1). Phylogenetic tree was drawn with MEGA 7.1 program show that more similarity among *Aspergillus* *niger* and low similarity with *A.* *niger* AHBR5 (Fig. 2).

### Ochratoxigenic potential of *A. niger*

Our results indicated that all the tested *A.* *niger* isolates had ability to produce ochratoxins by using flourometric method with variable levels (2.6–3.2 ppb) with the highest reading recorded by *A.* *niger*-27 recovered from coconuts as shown in Table 2.

**Table 2:** Ochratoxigenic potential of *A.* *niger* isolates.

Fungal isolates	Source	Accession number	Ochratoxins level
*A.* *nige*r-26	Almond-5	MW029470	2.7
*A.* *nige*r-27	Coconut-9	MW029471	3.2
*A* *nige*r-28	Almond-16	MW029472	2.6
*A.* *nige*r-29	Peanut-2	MW029473	3.1
*A.* *nige*r-30	Peanut-13	MW029474	2.8

### Detection of ochratoxins biosynthesis genes

**Figure 3:**
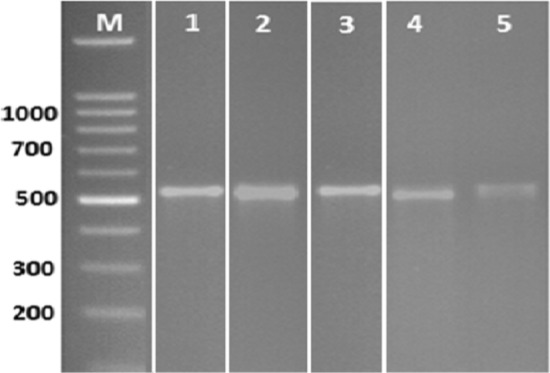
PCR amplification of *Aopks* genes (549 bp) for *A.* *niger* isolates. Whereas, (1)  *A.* *niger-*26 and (2)  *A.* *niger-*27*,* (3)  *A.* *niger-*28*,* (4)  *A.* *niger-*29 and (5)  *A.* *niger-*30. Sample lanes from different gels have been juxtaposed together in this figure.

Polymerase chain reaction (PCR) was applied using two sets of primer for gene involved in ochratoxin biosynthetic pathway. Bands of the fragments of *Aopks* gene can be visualized in all tested *A.* *niger* isolates at 549 bp (Fig. 3).

### Preliminary screening of *A. niger* isolates for lipase production

The tested *A.* *niger* isolates had ability to produce lipase enzyme in solid medium containing tween 80 with enzyme activity index (EAI) ranging from 2.02 to 3.28 as summarized in Table (3). White precipitate diameter was between 11.5 ± 0.5 and 21.8 ± 7.42 mm. *A.* *niger*-29 showed the highest diameter 21.8 ± 7.42 mm and the lowest was observed in *A.* *niger*-27 with 11.5 ± 0.5 mm.

### Assay of lipase enzyme

Lipase activity was determined in liquid medium by using trimetric method showed that *A.* *niger*-26 obtained from almond recorded the highest lipase activity (0.6 ± 0.1 U/ml-min) followed by *A.* *niger*-30 (0.3 ± 0.1 U/ml-min), *A.* *niger*-28 and *A.* *niger*-29 with the same reading (0.233 ± 0.11547 U/ml-min) and *A.* *niger*-27 was the least (0.2 ± 0.1 U/ml-min) (Table 3).

**Table 3:** Lipolytic activity of *A.* *niger* isolates.

Fungal isolates	Clear zone diameter (mm)	Enzyme activity index (EAI)	Lipase activity (U/ml-min)
*A.* *niger*-26	14 ± 2.08	3.07	0.6 ± 0.1*
*A.* *niger*-27	11.5 ± 0.5	2.78	0.2 ± 0.1*
*A*. *niger*-28	13.8 ± 1.04	3.11	0.233 ± 0.11547*
*A*. *niger*-29	12.5 ± 0.87	3.28	0.233 ± 0.11547*
*A.* *niger*-30	21.8 ± 7.42	2.02	0.3 ± 0*

*Means significant value in comparison with control with LSD at 0.05 was 0.16 for *A.* *niger*-26 and *A.niger*-29, 0 for *A.* *niger*-27 and *A.* *niger*-30 and 2.44 for *A.* *niger*-28. Values expressed as mean ± Standard deviation.

### Detection of *A. niger lip2* gene

**Figure 4:**
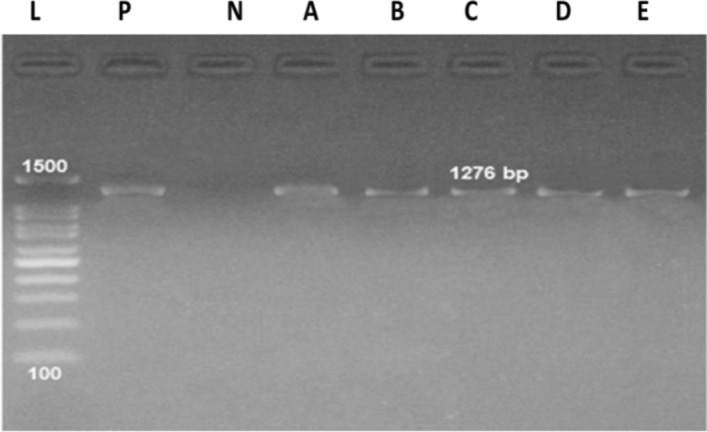
PCR amplification of *Lip2* genes (1276 bp) for *A.* *niger* isolates. whereas, **(A)**  *A.* *niger-*29, **(B)**  *A.* *niger-*26*,* **(C )** *A.* *niger-*28*,* **(D)** *A.* *niger-*27 and **(E)**  *A.* *niger-*30.

PCR was performed for *Lip2* gene detection in the tested *A.* *niger* isolates using two sets of primers. *Lip2* gene was detected at 1276 bp in all the *A.* *niger* isolates (Fig. 4).

### Studying the virulence properties of target human pathogens in presence of crude lipase obtained from *A. niger*

In the current study, we extended the utility of using crude lipase from *A.* *niger* to explore its potential as antibacterial against some human pathogens. This was performed using INT reduction assay. Results exhibited an excellent effect of crude lipase against both Gram negative and Gram positive tested strains. Where MIC ranged from 10 to 20 µl/100 µl and MBC from 20 to 40 for *Escherichia* *coli*, *Proteus* *mirabilis*, *Pseudomonas* *aeruginosa*, and Methicillin-resistant *Staphylococcus* *aureus* (MRSA) Table 4.

**Table 4:** Antibacterial efficacy of crude lipase against some human pathogens.

Tested pathogens	Antibacterial efficacy/100 µl	
	MIC	MBC
*Escherichia* *coli*	10 µl	20 µl
*Pseudomonas* *aeruginosa*	20 µl	20 µl
*Proteus* *mirabilis*	10 µl	20 µl
*Staphylococcus* *aureus* (MRSA)	30 µl	40 µl

### Antibiofilm activity lipase enzyme

**Figure 5:**
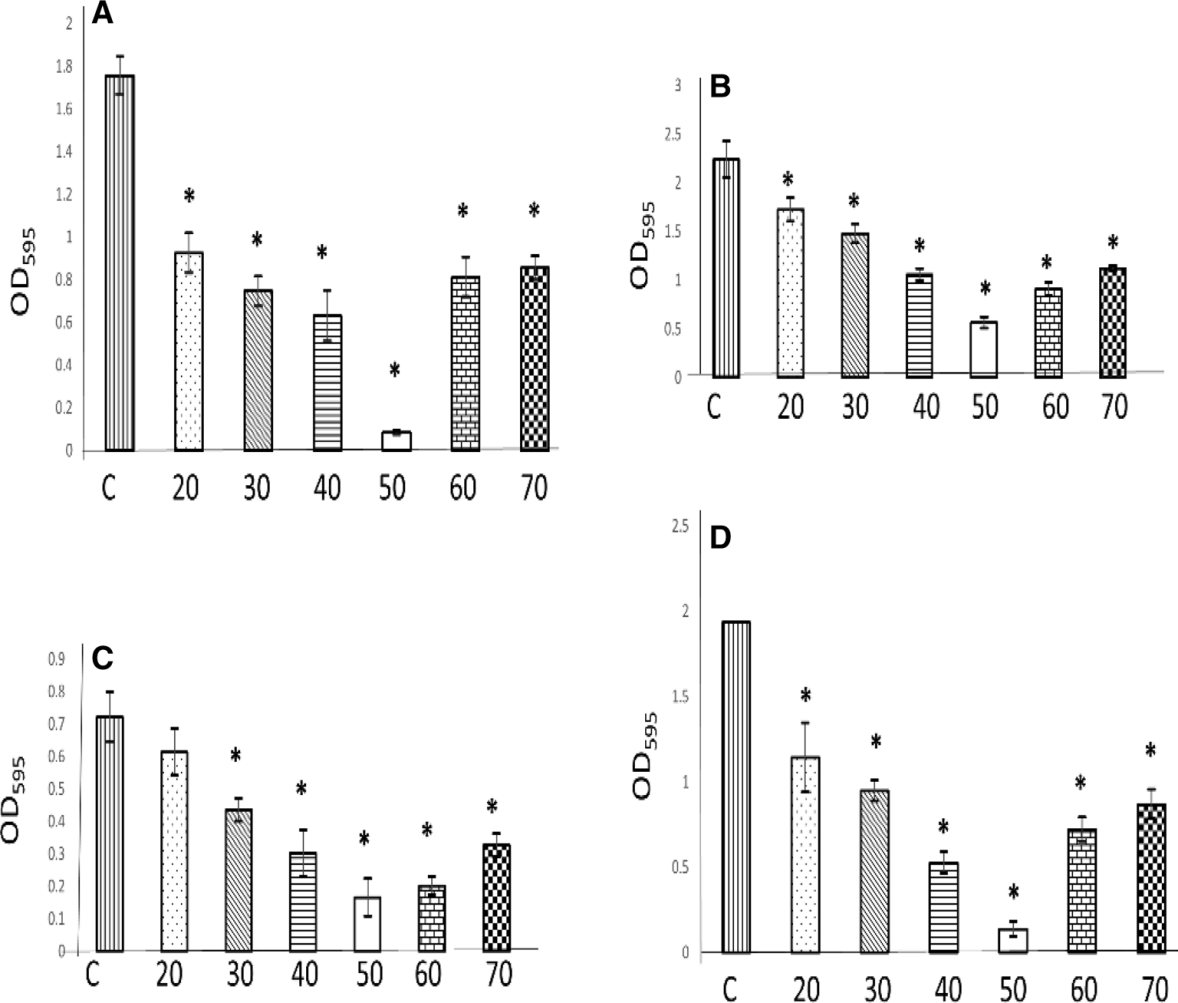
Antibiofilm activity of crude lipase produced by *A.* *niger* isolated from nuts against some human pathogenic bacteria. **(A)** *Escherichia* *coli*; **(B)** *Pseudomonas* *aeruginosa*; **(C)** *Proteus* *mirabilis*; **(D)** methicillin-resistant *Staphylococcus* *aureus* (MRSA). C: control (amount of biofilm of the tested strains). 20, 30, 40, 50, 60, and 70 µl: added volumes of crude lipase for determination the optimum volume in inhibiting biofilm. Shown are the averages from at least three independent measurements. The error bars indicate the standard deviations. Asterisk: means values are highly significant compared with control.

In our study crude lipase from *A.* *niger* MW029470 was examined as antibiofilm agent against four human pathogens by spectrophotometric methods. The ability of the four tested human pathogens *Escherichia* *coli*, *Pseudomonas* *aeruginosa*, *Proteus* *mirabilis* and Methicillin-resistant *Staphylococcus* *aureus* (MRSA) to form biofilm were confirmed before treatment with lipase as shown in Fig. 5A–D{C}, respectively. The results exhibited significant inhibition for biofilm formation in the four tested pathogens. The highest significant percentages of inhibition were 95.3, 74.9, 77.1 and 93.6 for *Escherichia* *coli*, *Pseudomonas* *aeruginosa*, *Proteus* *mirabilis*, and Methicillin-resistant *Staphylococcus* *aureus* (MRSA) Fig. 5A–D{50}, respectively.

### Scanning electron microscopy (SEM)

**Figure 6:**
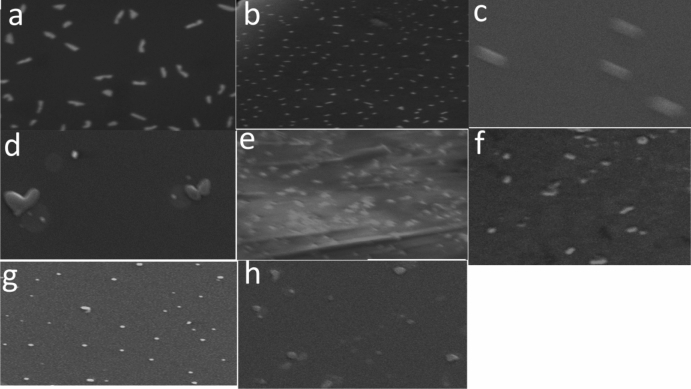
Scanning electron microscopy micrographs of treated bacteria with crude lipase. **(a,c,e,g)** Untreated *Escherichia* *coli*, *Proteus* *mirabilis*, *Pseudomonas* *aeruginosa* and methicillin-resistant *Staphylococcus* *aureus* (MRSA), respectively (control). **(b,d,f,h)** Treated *Escherichia* *coli*, *Proteus* *mirabilis*, *Pseudomonas* *aeruginosa* and methicillin-resistant *Staphylococcus* *aureus* (MRSA) with crude lipase, respectively (treatments).

**Figure 7:**
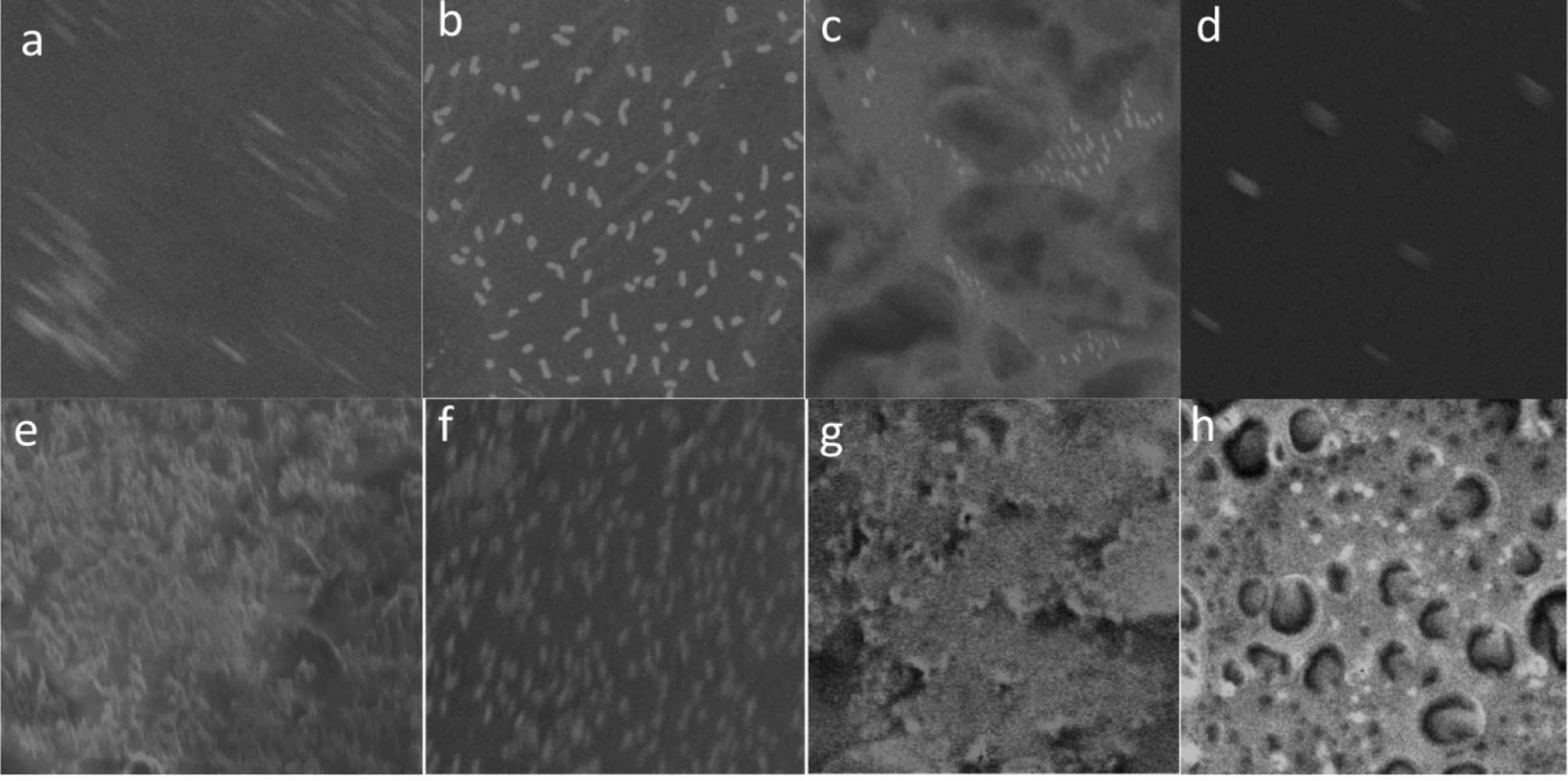
Scanning electron microscopy micrographs of biofilm structure. **(a,c,e,g)** Biofilm formation by untreated *Escherichia* *coli*, *Proteus* *mirabilis*, *Pseudomonas* *aeruginosa* and methicillin-resistant *Staphylococcus* *aureus* (MRSA), respectively (control). **(b,d,f,h)** Biofilm formation by treated *Escherichia* *coli*, *Proteus* *mirabilis*, *Pseudomonas* *aeruginosa* and methicillin-resistant *Staphylococcus* *aureus* (MRSA) with crude lipase, respectively (treatments).

Results of SEM were revealed in Figs. 6 and 7. For antibacterial efficacy of crude lipase for the tested bacteria, the micrographs showing that some cells shorten and getting smaller such as *Escherichia* *coli* (Fig. 6a,b). Other cells were curved and divided like *Proteus* *mirabilis* (Fig. 6c,d). Cells of *Pseudomonas* *aeruginosa* (Fig. 6e,f) distortion occur in cell shape to spherical instead of bacillus. Finally, cells of Methicillin-resistant *Staphylococcus* *aureus* (MRSA) that begin to swell up with irregular spherical shape Fig. 6g,h.

SEM micrographs for biofilm structure revealed that, in control, there are typically heterogeneous distributions of biofilm with higher number of adhered cells also cells arranged in the form of aggregates or simply as individualized cells without slimy material in their vicinity (Fig. 7a,c,e,g). In contrast, to treatment with crude lipase where micrographs showing a uniform layer of cells with negligible clumping (Fig. 7b,d,f,h**).**

## Discussion

Nuts and dried fruits are healthful foods that protect human body from many chronic diseases. Their high nutritional value makes them a suitable medium for fungal contamination. In the current study, peanut were the highest contaminated samples this may be due to the high moisture content of peanut samples in harmony with Ismail^28^, who reported that peanut samples were highly deteriorated with fungi than coconut. *Aspergillus* was the most prevalent genus followed by *Penicillium*. This were previously confirmed by Khosravi et al.^29^, who showed that *Aspergillus* followed by *Penicillium* were the most frequent genera deteriorated 60 samples of nuts. From the above genera *A.* *niger*, *A.* *flavus*, *P.* *chrysogenum* and *P.* *oxalicum* were the most frequent species (Table 1) and these results were previously obtained by Ismail^28,30–33^. In contrast, *Aspergillus* section Flavi was the highest recorded in peanuts seeds followed by *Aspergillus* section Nigri and *Aspergillus* section Circumdati was the least^34^. Past study by Tournas et al^5^ found the same results that *A.* *niger* followed by *Penicillium* were the most common mold in nuts and dried fruits. *Rhizopus* *stolonifer* was isolated from peanuts, almonds and raisins in high and moderate frequency of occurrence (Table 1). In a study by Abdulla^35^, reported that *Aspergillus*, *Rhizopus* and *Penicillium* genera were more frequently detected than other genera of fungi in nuts.

The molecular identification of the tested *A.* *niger* confirmed the morphological identity and more similarity among *A.* *niger* isolates was observed and low similarity with *A.* *niger* AHBR5 except *A.* *niger*-27 (Figs. 1, 2) and the obtained results in agreement with Perrone et al^36,37^. All the tested *A.* *niger* isolates were ochratoxin producers with variable readings by using fluorometric method (Table 2). In past investigation by Al-Sheikh^38^ confirmed that 57% and 60% of *A.* *niger* and *A.* *carbonarius*, respectively deteriorated peanut were ochratoxin producers. Magonli et al^39^, demonstrated that 32% of *Aspergillus* section Nigri obtained from peanut seeds in Argentinean had ability to produce ochratoxin A. Alhussaini^30^, found that 33.3% of *Aspergillus* section Nigri biserriate and one isolate of uniserriate isolated from nuts were ochratoxin A producers. The tested *A.* *niger* isolates recovered from baby foods recorded positive results for ochratoxins production^40^. Our obtained results were in-disagreement with past study by Palumbo & O’Keeffe^41^, reported that all the tested 171 isolates of *Aspergillus* section Nigri isolated from almonds showed negative results for ochratoxin A production. In this study, ochratoxin biosynthesis gene *Aopks* was detected in all the tested isolates at 549 bp (Fig. 3). The obtained results in harmony with Massi et al.^42^, who reported that *pks* gene was detected in all *Aspergillus* *niger* positive ochratoxgenic strains isolated from Brazilian foods amongst, nuts and dried fruits. *Aopks* genes were detected at 549 bp in *A.* *niger* isolates that had ability to produce ochratoxins^43, 44^. All tested *A.* *niger* and *A.* *tubingensis* isolated from beef showed positive results for the presence of *pks* genes^45^. The selected *A.* *niger* isolates were lipase producers qualitatively on Tween 80 solid medium and quantitatively by using trimetric titration method with the highest activity recorded by *A.* *niger*-26 isolated from almond (Table 3)*.* *A.* *niger* is well-recognized to be the best producer of lipase enzyme and is favored in many industrial processes^46, 47^. *A.* *niger*, *Fusarium* *oxysporum* and *Nectria* *haematococca* isolated from beef luncheon were the highest lipase producers^48^. Rai et al.^49^, isolated lipase producer *A.* *niger* from some oil contaminated soil samples. Earlier studies also, confirmed that *A.* *niger* was the highest lipase producing strain^50–54^. Putri et al.^12^, optimized the production of lipase by *A.* *niger* by using agro-waste and revealed that 1% olive oil was the highest inducer, yielding dry lipase extract with highest activity unit (176 U/ml enzyme). *Lip2* gene was visualized at 1276 bp in all the tested *A.* *niger* isolates (Fig. 4). Yang et al.^55^, reported *Lip2* gene a novel lipase gene cloned from *A.* *niger*. Lipase exhibits antibacterial activity against *Escherichia* *coli*, *Proteus* *mirabilis* and *Pseudomonas* *aeruginosa* with MBC of 20 µl/100 µl (Table 4). This may be due to that lipase acting on the lipopolysaccharide of Gram negative cell wall as well as the esters of exopolysaccharide present in the biofilm. Furthermore, lipolytic enzyme acts on a lipid substrate Such as phospholipids and other hydrophobic molecules, to hydrolyze or esterify a bond^16^. Lipases are esterases capable of hydrolyzing any ester bond. They act on the lipoprotein, lipopolysaccharide and phospholipids which surrounds the peptidoglycan layer leading to the hydrolysis of the lipid bilayer. The lipopolysaccharide complex is an endotoxin present on the outer membrane of the cell wall and this toxicity leads to a wide spectrum of nonspecific pathophysiological reactions including fever, changes in white blood cell counts, disseminated intravascular coagulation, hypotension, shock and death. When lipase works on lipid A, the chances of infection are reduced^56^. In most of the Gram positive bacteria, lipoteichoic acids are present and the lipid tail present here plays a major role in the bacterial attachment. There is a possibility for the lipase to act on this lipid tail thereby preventing its adherence to a surface^16^. Our results confirmed antibacterial activity of lipase on Gram positive bacteria (MRSA) with MBC of 40 µl/100 µl (Table 4). Bacterial biofilms pose a great threat to human life not only because they involved in a lot of chronic infectious human diseases but also, they highly resistant to different antimicrobial agents. This generates a strong demand for finding suitable anti biofilm agents^57^. Bacterial biofilms are common populations of bacterial cells surrounded by a self-produced matrix of extracellular polymeric substances (EPS) ^58^. EPS surrounding mixture include various exopolysaccharides, lipids, secreted proteins some of which can form amyloid fibers and extracellular DNA^59^. Most of the antimicrobial agents fail to penetrate the biofilm owing to the presence of EPS which acts as a barrier protecting the bacterial cells within the biofilm. So, the remedy will be the use of compounds that able to degrade the biofilm EPS. Enzymes have been recognized to be effective for the degradation of the biofilms EPS^60,61^. Plants contain various anti-biofilm compounds, as they have to prevent bacterial growth on their surfaces^62,63^. Since lipase, an esterase, is a hydrolyzing enzyme, it is having the ability to act on the EPS produced by the organisms^64^, by degrading protein components and the high molecular weight lipid of the biofilm^65^. In the current investigation crude lipase, was examined as antibiofilm agent against four human pathogens by spectrophotometric methods. The highest significant percentages of inhibition were 95.3, 74.9, 77.1 and 93.6 for *Escherichia* *coli*, *Pseudomonas* *aeruginosa*, *Proteus* *mirabilis* and Methicillin-resistant *Staphylococcus* *aureus* (MRSA), respectively. Although, all added volumes of lipase significantly inhibited biofilm formation, the suitable volume that gives highest inhibition percentage was 50 µl (Fig. 5). Scanning electron microscopy (SEM) has been used widely for qualitative observation of biofilm before and after treatments, biofilm disturbance due to its high resolution and is usually applied in biological assays of biofilm removal effectiveness also antimicrobial treatments^66–68^. Results of SEM confirmed antbacterial and antibiofilm properties of crude lipase against the tested human pathogens.

In conclusion, Peanuts were the highest contaminated samples among the tested types. *A.* *niger* was the most isolated species from nuts. All the selected *A.* *niger* isolates were lipase producers with highest enzyme activity was recorded by *A.* *niger* MW029470 and showed positive results for the presence of *Lip* *2* gene. Crude lipase from *A.* *niger* MW029470 showed highly inhibition of the tested pathogens growth with MBC of 20 to 40 µl/100 µl and significantly inhibited biofilm formation of 4 biofilm former human bacterial pathogens. The significant percentages of inhibition were 95.3, 74.9, 77.1, and 93.6 for *Escherichia* *coli*, *Pseudomonas* *aeruginosa*, *Proteus* *mirabilis* and Methicillin-resistant *Staphylococcus* *aureus* (MRSA), respectively.

## Materials and methods

### Collection of nuts samples

Eighty samples of peanut, almond, coconut and raisin (20 samples of each type) were purchased from different supermarkets at Qena Governorate, Egypt. All samples were kept in a refrigerator until mycological analysis.

### Isolation of fungi

The modified method described by Tournas et al.^5^ was employed for isolation of mycobiota contaminating nuts. A known weight of each sample was blended with 90 ml of 0.1% peptone in blender jar under aseptic conditions for minute. Serial dilutions were made to obtain the suitable one. One ml of the suitable dilution was poured in sterilized petri plate followed by 20 ml of rose Bengal chloramphenicol agar (RBCA) medium containing g/l (peptone; 5, glucose; 10, kH_2_PO_4_; 1, MgSO_4_. 7H_2_O; 0.5, rose Bengal; 0.05, chloramphenicol; 0.1, and agar 15.5). Triplicates of each sample were prepared. Plates were incubated for a week at 28 °C. The developed fungal colonies were counted, examined and identified (based on macro- and microscopic features)^69^.

### Sequence analysis of 5.8S-ITS region

ITS1 and ITS2 regions together with 5.8S gene in rDNA from *A.* *niger* strains were amplified as designed by Hermosa et al.^70^. The purified bands were determined using the sequencer Gene analyzer 3121 in Scientific Research Center, Biotechnology and Genetic Engineering Unit, Taif University, KSA. The realized sequence was aligned using Molecular Evolutionary Genetics Analysis (MEGA) version 5.10. Then, a consensus sequence was generated from each alignment made. The sequencing data were compared against the Gene Bank database (http://www.ncbi.nlm.nih.gov/BLAST/), where a nucleotide blast program was chosen to identify the homology between the PCR fragments and the sequences on the Gene Bank database.

### Accessions numbers

Sequences were deposited in GenBank under accession numbers MW029470-MW029474.

### Ochratoxins production by *A. niger* isolates

Five isolates of *Aspergillus* *niger* with the highest number of colonies and high frequency of occurrence were tested for their ability to produce ochratoxins by cultivation in conical flasks containing 50 ml of yeast extract sucrose (YES) liquid medium with composition sucrose, 40 g, yeast extract 20 g, and distilled water, 1000 m1. Incubation the flasks at 28 °C for fifteen days^71^. Filtration through a fluted filter paper (Whatman 2 V, Whatmanplc, Middlesex,UK). Total ochratoxins were determined according to the method mentioned by El-Dawy et al.^44^ in 10 ml fungal filtrate by adding 90 ml (methanol: water) (80:20 v/v) and the filtrate was diluted (1:4) with distilled water and re-filtered through a glass-fiber filter paper. Ten milliliters of the glass-fiber filtrate were placed on Ochra test WB SR Column (VICAM, Watertown, MA, USA) and allowed to elute at 1–2 drops/s. The columns were washed twice with 10 ml of distilled water, and ochratoxins were eluted from the column by adding 1 ml of methanol HPLC and delivered in clean cuvette. 1.5 ml ochratoxin eluting agent was added and the total ochratoxins concentration were measured after calibration VICAMSeries-4 fluorometer set at 360 nm excitation and 450 nm emissions^72^.

### Molecular detection of ochratoxin-producing genes

DNA extraction and purification were performed using DNA Promega Kit DNeasy Blood & Tissue (Valencia, CA, USA). Two published primers were used for the specific detection of ochratoxin biosynthesis genes. The sequence of primers was as following: *Aopks*-F '5-CAGACCATCGACACTGCATGC-'3, *Aopks*-R '5- CTGGCGTTCCAGTACCATGAG-'3^73^. The 630 bp fragments were amplified, PCR was performed in a reaction volume of 25 μl according to Hussein et al.^74^ The reactions were done in a C1000.

Thermo Cycler BioRad, Germany with initial denaturizing at 94 °C for 5 min, followed by 30 cycles of 1 min. at 94 °C, 1 min. at 58 °C and extension at 72 °C for 1 min^43^, then final step as extension at 72 °C for 10 min. PCR products were checked on a 1.3% agarose gel and stained with ethidium bromide.

### Screening *A. niger* isolates for lipase production

Tween 80 agar plate was used for screening the tested isolates for lipase production containing (g/l peptone, 15; NaCl, 5; CaCl_2_, 1; tween 80, 10 and agar, 15) and pH of the media was adjusted to 7. 250 µl of fungal spore suspension (8 × 10^7^ spores/ ml) was inoculated to 8 mm cavity on the media and incubated at 28 °C for 4 days. Appearance of white precipitate around the fungal colony indicates the ability to produce lipase enzyme^49^.

### Quantitative estimation of lipase

Trimetric method was applied for assay lipase^75^ with some modification. Two disks (8 mm) of tested isolates were inoculated to minimal medium containing (g/l 1 yeast extract, 1 KCl, 1 MgSO_4_.7H_2_O), pH 6 and supplemented with (1% v/v tween 80) and incubated at 30 °C on shaker incubator at 150 rpm for 3 days. 1.5 ml suspension from liquid medium was centrifuged at 3000 rpm for 10 min. Assay mixture containing 500 µl 0.1 M phosphate buffer (pH 6.8), 500 µl tween 80 and 250 µl crude enzyme was incubated at 37 °C for 20 min. Three ml of acetone: ethanol (1:1) was added to stop the reaction. The liberated fatty acids were titrated with 1 N NaOH solution with bromothymol blue indicator. In control 250 µl distilled water was added instead of crude enzyme. Lipase activity (U/ml-min) was calculated from the following equation:

$${\text{Lipase activity}}\, = \,\left( {{\text{T}} - {\text{C}}} \right)\, \times \,{\text{N}}\, \times \,{\text{df}}/({\text{t}}\, \times \,{\text{v}}).$$

T is the titration volume, C is the control, N is the normality of NaOH, df is the volume of assay/volume of enzyme, t is the incubation time, v is the sample.

### Detection of *lip2* gene in *Aspergillus niger* isolates

*Aspergillus* *niger* *lip2* gene was detected by using 2 documented primers. The sequences of primers were as following: *P1* (5’-CTCAAGAGTATCCTGCACTG-3’) and *P2* (5’-CTGAACCTTCCTTGGGATAG-3’)^55^. Twenty-five μl as volume was used for PCR reaction by mixing 12.5 µl of EmeraldAmp Max PCR Master Mix (Takara, Japan), 1 µl of each primer, 6 µl of DNA template and 4.5 µl of water was added to make the volume up to 25 μl. Applied biosystem 2720 thermal cycler was used for performing the reaction with initial denaturizing at 94 °C for 5 min, followed by 35 cycles of 30 s. at 94 °C, 50 s. as annealing temperature at 59 °C and 1 s. at 72 °C. Ten minutes at 72 °C was used as the final extension. The PCR products were checked on 1.5% agarose gel in 1 × TBE buffer. A gelpilot 100 bp plus DNA Ladder was used to determine the fragment sizes. The gel was photographed by a gel documentation system. Data was analyzed through computer software.

### Studying the virulence properties of target human pathogens in presence of crude lipase obtained from *A. niger*

#### Determination of minimum inhibitory concentration (MIC)

MIC was evaluated by *p*-iodonitrotetrazolium violet choloride (INT) formazon assay (0.2 mg/ml, SIGMA-ALDRICH). Overnight cultures of *Escherichia* *coli*, *Pseudomonas* *aeruginosa*, *Proteus* *mirabilis*, and Methicillin-resistant *Staphylococcus* *aureus* (MRSA) were adjusted to OD_595_ of 0.01 into tryptic soy broth (TSB). 100 µl of each freshly prepared bacterial culture were placed into 96-well plates plus different volumes of crude lipase (10–100 µl, 8 replicates were made for each volume in complete raw). After 24 h incubation at 37 °C, to confirm bacterial growth suppression and deficiency of metabolic activity, 40 µl INT was added to the microplate wells and re-incubated at 37 °C for 30 min. The MIC in the INT assay was defined as the lowest concentration that suppressed bacterial growth and prevented color change^62,76–78^.

### Determination of minimum bactericidal concentration (MBC)

The bactericidal efficacy was defined as a 99.9% decrease in CFU (3 logs) in the initial inoculum during 24 h of incubation. The MBC was determined by inoculating sterilized tryptic soy agar (TSA) fresh plates with 50 µl from each well of overnight MIC plates. Viable colonies were counted after 24 h at 37 °C. The limit of detection for this assay was 10 cfu ml^−1^^62,79^.

### Static biofilm assay

The tested bacterial strains {*Escherichia* *coli*, *Pseudomonas* *aeruginosa*, *Proteus* *mirabilis* and Methicillin-resistant *Staphylococcus* *aureus* (MRSA)} were obtained kindly from international Luxor hospital. The ability of the verified pathogens for biofilm formation was determined using 96-well polystyrene plates^80^. Bacterial strains were subcultured on tryptic soy agar for 24 h at 37 °C, suspended in tryptic soy broth and adjusted to an OD_595_ of 0.02. 130 µl of each adjusted isolate culture were put in the microtitre plate (U bottom, Sterilin) at 37 °C for 24 h. After incubation the wells were washed six times with distilled water, Furthermore, the wells were stained with 0.1% crystal violet for 10 min. the wells were again washed with distilled water (4 times) to remove excess stain^81^. Finally, the wells were destained by 210 µl of ethanol 96% and the OD_595_ was read using infiniteF50 Robotic (Ostrich) microplate plate to quantify the amount of biofilm.

### Antibiofilm efficacy of crude lipase

The effect of crude lipase enzyme with the highest activity from *A.* *niger* MW029470 free of ochratoxin after 3 days of incubation as antibiofilm against four human pathogenic biofilm former bacteria was done by spectrophotometric methods. Different volumes (20, 30, 40, 50, 60, and 70 µl) were added to 130 µl of the tested pathogens at OD_595_ of 0.02 after 24 h incubation at 37 °C for allowing biofilm formation. The plates then incubated for further 24 h and then stained with crystal violet as described previously^27^.

### Scanning electron microscopy (SEM) analysis of antibacterial and antibiofilm efficacy of crude lipase

Preparation of samples for antibacterial was performed as described by Wang et al.^82^. While for biofilm was done as described by Kong et al.^83^ and Chin et al.^84^ with little modifications. Biofilms were allowed to form on the slides at 37 °C for 24 h alone (control) and after treatment with crude lipase following which, the samples were fixed in 4% (v/v) glutaraldehyde in 0.05 M phosphate buffer (pH 7.0) at 4 °C for 12 h. Subsequently, the samples were washed three times in phosphate buffer, dehydrated through a graded ethanol series, dried in a critical-point drying apparatus with liquid carbon dioxide; slides coated with gold and viewed using (JEOL JSM-5500LV, Japan)**.**

### Statistical analysis

The variability degree of results was expressed in form of means ± standard deviation (mean ± SD) based on triplicates determinations (n = 3 for replicate plates). The data were statistically analyzed by one-way ANOVA analysis and compared using the least significant difference (LSD) test at 0.05 (*) levels. It was done to compare between control and treatments.

## References

[1] Carughi A, Feeney MJ, Kris-Etherton P, Fulgoni V, Kendall CW, Bulló M, Webb D (2015). Pairing nuts and dried fruit for cardiometabolic health. Nutr. J..

[2] Li M, Fan Y, Zhang X, Hou W, Tang Z (2014). Fruit and vegetable intake and risk of type 2 diabetes mellitus: Meta-analysis of prospective cohort studies. BMJ Open.

[3] Hemandez-Alonso P, Salas-Salvado J, Baldrich-Mora M, Juanola-Falgarona M, Bullo M (2014). Beneficial effect of pistachio consumption on glucose metabolism, insulin resistance, inflammation, and related metabolic risk markers: A randomized clinical trial. Diabetes Care.

[4] Weidenborner M (2001). Pine nuts: The mycobiota and potential mycotoxins. Can. J. Microbiol..

[5] Tournas VH, Niazi NS, Kohn JS (2015). Fungal presence in selected tree nuts and dried fruits. Microbiol. Insights..

[6] Agriopoulou S, Koliadima A, Karaiskakis G, Kapolos J (2016). Kinetic study of aflatoxins degradation in the presence of ozone. Food Control.

[7] Benedict K, Chiller TM, Mody RK (2016). Invasive fungal infections acquired from contaminated food or nutritional supplements. Foodborne Path. Dis..

[8] Pfohl-Leszkowicz A (2009). Ochratoxin A and aristolochic acid involvement in nephropathies and associated urothelial tract tumours. Arh. Hig. Rada. Toksikol..

[9] Wafa, E. W., Yahya, R. S., Sobh, M. A., Eraky, I., El Baz, H., El Gayar, H. A. M., Betbeder, A. M. & Creppy, E. E. Human ochratoxicosis and nephropathy in Egypt: A preliminary study. *Hum.**Exp.**Toxicol*. **17**, 124–129 (1998). 10.1177/096032719801700207 9506263

[10] Schwartz GG (2002). Hypothesis: Does ochratoxin A cause testicular cancer?. Cancer Causes Control..

[11] Singh AK, Mukhopadhyay M (2012). Overview of fungal lipase: A review. Appl. Biochem. Biotechnol..

[12] Putri, D. N., Khootama, A., Perdani, M. S., Utami, T. S. & Hermansyah, H. Optamization of Aspergillus niger lipase production by solid state fermentation of agro-industrial waste. *Energy**Rep*. **6**, 331–335 10.1016/j.egyr.2019.08.064 (2020).

[13] Hasan F, Shah AA, Hameed A (2006). Industrial applications of microbial lipases. Enzyme Microb. Technol..

[14] Kristensen, J.B., Meyer, R.L., Laursen, B.S., Shipovskov, S., Besenbacher, F. & et al. Antifouling enzymes and the biochemistry of marine settlement. *Biotechnol*. *Adv*. **26**, 471–481 (2008). 10.1016/j.biotechadv.2008.05.005 18619758

[15] Carvajal, J.C., McDaniel, C.S. & Wales, M.E. Enzymatic antimicrobial and antifouling coating and polymeric materials. In *US**Patent/0238811**A1* (2009).

[16] Prabhawathi V, Boobalan T, Sivakumar PM, Doble M (2014). Antibiofilm properties of interfacially active lipase immobilized porous polycaprolactam prepared by LB technique. PLoS ONE.

[17] Achchi NIB, Khan F, Kim YM (2020). Inhibition of virulence factors and biofilm formation of *Acinetobacter**baumannii* by naturally-derived and synthetic drugs. Curr. Drug Targets..

[18] Khan F, Tabassum N, Pham DTN, Oloketuyi SF, Kim YM (2020). Molecules involved in motility regulation in *Escherichia**coli* cells: A review. Biofouling.

[19] Khan F, Khan MM, Kim YM (2018). Recent progress and future perspectives of antibiofilm drugs immobilized on nanomaterials. Curr. Pharm. Biotechnol..

[20] Khan F, Oloketuyi SF, Kim YM (2019). Diversity of bacteria and bacterial products as antibiofilm and antiquorum sensing drugs against pathogenic bacteria. Curr. Drug Targets.

[21] Khan F, Pham DTN, Oloketuyi SF, Manivasagan P, Oh J, Kim YM (2020). Chitosan and their derivatives: Antibiofilm drugs against pathogenic bacteria. Colloids Surf. B Biointerfaces.

[22] Mulat M, Pandita A, Khan F (2019). Medicinal plant compounds for combating the multi-drug resistant pathogenic bacteria: A review. Curr. Pharm. Biotechnol..

[23] Khan F, Lee JW, Javaid A, Park SK, Kim YM (2020). Inhibition of biofilm and virulence properties of Pseudomonas aeruginosa by sub-inhibitory concentrations of aminoglycosides. Microb. Pathog..

[24] Khan F, Pham DTN, Oloketuyi SF, Kim YM (2020). Regulation and controlling the motility properties of *Pseudomonas**aeruginosa*. Appl. Microbial. Biotechnol..

[25] Khan, F., Tabassum, N., Anand, R. & Kim, Y. M. Motility of *Vibrio* spp.: Regulation and controlling strategies. *Appl.**Microbiol.**Biotechnol.* 1–22 10.1007/s00253-020-10794-7 (2020). 10.1007/s00253-020-10794-7 32816086

[26] Xavier JB, Picioreanu C, Rani SA, Van Loosdrecht MCM, Stewart PS (2005). Biofilm control strategies based on enzymatic disruption of the extracellular polymeric substance matrix_a modeling study. Microbiology.

[27] Elamary, R., & Salem, W.M. Optimizing and purifying extracellular amylase from soil bacteria to inhibit clinical biofilm-forming bacteria. *PeerJ* **8**, e10288 10.7717/peerj.10288 (2020). 10.7717/peerj.10288 PMC7643558 33194439

[28] Ismail MA (2001). Deterioration and spoilage of peanuts and desiccated coconuts from two sub-Saharan tropical east african countries due to the associated mycobiota and their degradative enzymes. Mycopathologia.

[29] Khosravi AR, Shokri H, Ziglari T (2007). Evaluation of fungal flora in some important nut products (pistachio, peanut, hazelnut and almond) in Tehran, Iran. Pak. J. Nutr..

[30] Alhussaini, M. S. Mycobiota and mycotoxins of nuts and some dried fruits from Saudi Arabia. *J.**Am.**Sci*. **8 (12)**, 525–534, http://www.jofamericanscience.org (2012).

[31] Kazemi A, Ostadrahim A, Ashrafnejad F, Sargheini N, Mahdavi R, Farshchian M, Mahluji S (2014). Mold contamination of untreated and roasted with salt nuts (walnuts, peanuts and pistachios) sold at markets of Tabriz, Iran. Jundishapur. J. Microbiol..

[32] Abbas, M., Naz, S. A., Shafigue, M., Jabeen, N. & Abbas, S. Fungal contamination in dried fruits and nuts: A possible source of mycosis and mycotoxicosis. *Pak.**J.**Bot.* **51(4)**, 1523–1529, 10.30848/PJB2019-4(31) (2019).

[33] Zohri AA, Abdel-Gawad KM (1993). Survey of mycoflora and mycotoxins of some dried fruits in Egypt. J. Basic Microbiol..

[34] Sultan Y, Magan N (2010). Mycotoxigenic fungi in peanuts from different geographic regions of Egypt. Mycotoxin Res..

[35] Abdulla NQF (2013). Evaluation of fungal flora and mycotoxin in some important nut products in Erbil local markets. Res. J. Environ. Earth Sci..

[36] Perrone G, Susca A, Epifani F, Mule G (2006). AFLP characterization of Southern Europe population of *Aspergillus* section *Nigri* from grapes. Int. J. Food Microbiol..

[37] Botton A, Ferrigo D, Scopel C, Causin R, Bonghi C, Ramina A (2008). A cDNA-AFLP approach to study ochratoxin A production in *Aspergillus**carbonarius*. Int. J. Food Microbiol..

[38] Al-Sheikh, H. M. LAMP-PCR detection of ochratoxigenic *Aspergillus* species collected from peanut kernel. *Genet.**Mol.**Res*. (GMR) **14(1)**, 634–644, 10.4238/2015.January.30.5 (2015). 10.4238/2015.January.30.5 25729999

[39] Magonli, C., Astoreca, A., Posone, M.L., Fernandez-Juri, M.G., Barberis, C. & Dalcero, A.M. Ochratoxin A and *Aspergillus* section Nigri in peanut seeds at different months of storage in Cordoba, Argentina. *Int.**J.**Food**Microbiol*. **1, 119(3)**, 213–8, 10.1016/j.ijfoodmicro.2007.07.056 (2007). 10.1016/j.ijfoodmicro.2007.07.056 17854935

[40] Yassein AS, El-Said AHM, El-Dawy EGA (2020). Biocontrol of toxigenic *Aspergillus* strains isolated from baby foods by essential oils. Flavour Fragr. J..

[41] Palumbo JD, O’Keeffe TL (2013). Distribution and mycotoxigenic potential of *Aspergillus* section Nigri species in naturally contaminated almonds. J. Food Prot..

[42] Massi, F. P., Sartori, D., Ferranti, L. deS., Imanaka, B. T., Taniwak, M. H. Vieira, M. L. C. & Fungaro, M. H. P. Prospecting for the incidence of genes involved in ochratoxin and fumonisin biosynthesis in Brazilian strains of *Aspergillus**niger* and *Aspergillus**welwitschiae*. *Int.**J.**Food**Microbiol*. **16(221)**, 19–28, 10.1016/j.ijfoodmicro.2016.01.010 (2016). 10.1016/j.ijfoodmicro.2016.01.010 26803270

[43] El‐Hamaky, A. M., Atef, A. H., El Yazeed, H. A. & Refai, M. K. Prevalence and detection of toxigenic *A.**flavus*, *A.**niger* and *A.**ochraceus* by traditional and molecular biology methods in feeds. *Int.**J.**Curr.**Res*. **8**, 25621–25633 (2016).

[44] El-Dawy EGA, Yassein AS, El-Said AHM (2019). Detection of mycobiota, aflatoxigenic and ochratoxigenic genes, and cytotoxic ability in spices. Food Sci. Nutr..

[45] Hussein MA, Gherbawy Y (2019). Genotypic identification of ochratoxigenic *Aspergilli* that contaminated beef luncheon and their protease activity. Rend. Lincei-Sci. Fis..

[46] Macris B, Kourentzi E, Hatzinkolaou DG (1996). Studies on localization and regulation of lipase production by *Aspergillus**niger*. Process Biochem..

[47] Mala JG, Edwinoliver NG, Kamini NR, Puvanakrishnan R (2007). Mixed substrate solid state fermentation for production and extraction of lipase from *Aspergillus**niger* MTCC 2594. J. Gen. Appl. Microbiol..

[48] Saleem A (2008). Effect of some food preservatives on lipolytic activity of beef luncheon fungi. Mycobiology..

[49] Rai, B., Shreshtha, A., Sharma, S. & Joshi, J. Screening, optimization and process scale up for pilot scale production of lipase by *Aspergillus**niger*. *Biomed.**Biotechnol*. **2(3)**, 54–59, 10.12691bb-2-33 (2014). https: //pubs.scieup.com/bb-2-3-3.

[50] Falony G, Armas JC, Mendoza JCD, Hernandez JLM (2006). Production of extracellular lipase from *Aspergillus**niger* by solid state fermentation. Food Technol. Biotechnol..

[51] Mahadik ND, Puntambekar US, Bastawde KB, Khire JM, Gokhale DV (2002). Production of acidic lipase by *Aspergillus**niger* in solid state fermentation. Proc. Biochem..

[52] Olama ZA, El-Sabaeny AH (1993). Lipase production by *Aspergillus**niger* under various growth conditions using a solid state fermentation. Microbiologia. (Madrid).

[53] Kamini NR, Mala JGS, Puvanakrishnan P (1999). Lipase production from *Aspergillus**niger* by solid state fermentation using gingelly oil cake. Process. Biochem..

[54] Mukhtar, H., Hanif, M., Ur-Rehman, A., Nawaz, A. & Ul-Haq, I. Studies on lipase production by *Aspergillus**niger* through solid state fermentation. *Pak.**J.**Bot*. **47(SI)**, 351–354 (2015).

[55] Yang J, Sun J, Yan Y (2010). lip2, a novel lipase gene cloned from *Aspergillus**niger* exhibits enzymatic characteristics distinct from its previously identified family member. Biotechnol. Lett..

[56] Davies D (2003). Understanding biofilm resistance to antibacterial agents. Nat. Rev. Drug Discov..

[57] Pruteanu M, Hernández Lobato JI, Stach T, Hengge R (2020). Common plant flavonoids prevent the assembly of amyloid curli fibres and can interfere with bacterial biofilm formation. Environ. Microbiol..

[58] Flemming HC, Wuertz S (2019). Bacteria and archaea on Earth and their abundance in biofilms. Nat. Rev. Microbiol..

[59] Flemming HC, Wingender J (2010). The biofilm matrix. Nat. Rev. Microbiol..

[60] Kalpana BJ, Aarthy S, Pandian SK (2012). Antibiofilm activity of α-amylase from *Bacillus**subtilis* S8–18 against biofilm forming human bacterial pathogens. Appl. Biochem. Biotechnol..

[61] Lequette Y, Boelsb G, Clarissea M, Faille C (2010). Using enzymes to remove biofilms of bacterial isolates sampled in the food-industry. Biofouling.

[62] Elamary RB, Albarakaty FM, Salem WM (2020). Efficacy of Acacia nilotica aqueous extract in treating biofilm-forming and multidrug resistant uropathogens isolated from patients with UTI syndrome. Sci. Rep..

[63] Silva NL, Zimmer KR, Macedo AJ, Trentin SD (2016). Plant natural products targeting bacterial virulence factors. Chem. Rev..

[64] Rosenberg M, Gutnick D, Rosenberg E (1980). Adherence of bacteria to hydrocarbons: A simple method for measuring cell-surface hydrophobicity. FEMS Microbiol. Lett..

[65] Reifsteck F, Wee S, Wilkinson BJ (1987). Hydrophobicity-hydrophilicity of staphylococci. J. Med. Microbiol..

[66] Whittaker C, Ridgway H, Olson BH (1984). Evaluation of cleaning strategies for removal of biofilms from reverse-osmosis membranes. Appl. Environ. Microbiol..

[67] Vickery K, Pajkos A, Cossart Y (2004). Removal of biofilm from endoscopes: Evaluation of detergent efficiency. Am. J. Infect. Control.

[68] Vyas N, Sammons RL, Addison O, Dehghani H, Walmsley AD (2016). A quantitative method to measure biofilm removal efficiency from complex biomaterial surfaces using SEM and image analysis. Sci. Rep..

[69] Domsch, K. H., Gams, W. & Anderson, T. H. *Compendium**of**Soil**Fungi,**Taxonomically**Revised**by**W.**Gams.* 672 (IHW, 2007). ‏

[70] Hermosa MR, Keck E, Chamorro I, Rubio B, Sanz L, Vizcaino JA, Grodona I, Monte E (2004). Genetic diversity shown in Trichoderma biocontrol isolates. Mycol. Res..

[71] Gabal MA, Hegazy SM, Nagwa YH (1994). Aflatoxin production by *Aspergillus**flavus* field isolates. Vet. Hum. Toxicol..

[72] Lewis L, Onsongo M, Njapau H, Schurz-Rogers H, Luber G, Kieszak S, Nyamongo J (2005). Aflatoxin contamination of commercial maize products during an outbreak of acute aflatoxicosis in eastern and central Kenya. Environ. Health Perspect..

[73] Reddy KV, Naveen K, Reddy IB (2013). Incidence and molecular detection of ochratoxigenic fungi from Indian cereal grains. Int. J. Pharm. Biol. Sci..

[74] Hussein, M. A., El-Said, A. H. M. & Yassein, A. S. Mycobiota associated with strawberry fruits, their mycotoxin potential and pectinase activity. *Mycology* **11(2)**, 158–166, 10.1080/21501203.2020.1759719 (2020). 10.1080/21501203.2020.1759719 PMC7448860 32923023

[75] Elegado, F., Legaspi, C. L., Paet, J. M., Querlibin, F., Tolentino, J. E., Vilela, J., Jr, A. P., Maloles, J. & Zarate, J. Screening, identification and optimization of extracellular lipase production of yeast (*Cryptococcus**flavescens*) isolated from a tree canopy fern in the mount Makiling forest reserve, Philippines. In *AIP**Conference**Proceedings* Vol. 2155 (1), 020029, https://doi.org/10.1063/1.5125533 (AIP Publishing LLC, 2019).

[76] Eloff JN (1998). A sensitive and quick microplate method to determine the minimal inhibitory concentration of plant extracts for bacteria. Planta. Med..

[77] Lall, N., Henley-Smith, C.J., De Canha, M.N., Oosthuizen, C.B. & Berrington, D. Viability reagent, presto blue, in comparison with other available reagents, utilized in cytotoxicity and antimicrobial assays. *Int.**J.**Microbiol*. (2013). 10.1155/2013/420601 PMC3638707 23653650

[78] Salem WM, El-Hamed DS, Sayed W, Elamary R (2017). Alterations in virulence and antibiotic resistant genes of multidrug-resistant *Salmonella**serovars* isolated from poultry: The bactericidal efficacy of *Allium**sativum*. Microb. Pathog..

[79] Sirelkhatim A, Mahmud S, Seeni A, Kaus NHM, Ann LC, Bakhori SKM, Hasan H, Mohamed M (2015). Review on zinc oxide nanoparticles: Antibacterial activity and toxicity mechanism. Nano-Micro. Lett..

[80] Khan F, Lee JW, Pham DTN, Lee JH, Kim HW, Kim YK, Kim YM (2020). Streptomycin mediated biofilm inhibition and suppression of virulence properties in *Pseudomonas**aeruginosa* PAO1. Appl. Microbiol. Biotechnol..

[81] Merritt, D.J., Turner, S.R., Commander, L.E. & Dixon, K.W. (eds). *Proceedings**of**the**Fifth**Australian**Workshop**on**Native**Seed**Biology**Brisbane,**Australia* (2005).

[82] Wang, J., Ma, M., Yang, J., Chen, L., Yu, P., Wang, J. & Zeng, Z. In vitro antibacterial activity and mechanism of monocaprylin against *Escherichia**coli* and *Staphylococcus**aureus*. *J.**Food**Prot*. **81(12)**, 1988–1996 (2018).‏ 10.4315/0362-028X.JFP-18-248 30461297

[83] Kong C, Chee CF, Richter K, Thomas N, Rahman NA, Nathan S (2018). Suppression of *Staphylococcus**aureus* biofilm formation and virulence by a benzimidazole derivative, UM-C162. Sci. Rep..

[84] Chin, C. Y., Hara, Y., Ghazali, A. K., Yap, S. J., Kong, C., Wong, Y. C., & Nathan, S. Global transcriptional analysis of *Burkholderia**pseudomallei* high and low biofilm producers reveals insights into biofilm production and virulence. *BMC**Genomics* **16(1)**, 471(2015).‏ 10.1186/s12864-015-1692-0 PMC4474458 26092034

